# One-step plastic stent placement using endoscopic ultrasound-guided hepaticogastrostomy without tract dilation in obstructive jaundice

**DOI:** 10.1055/a-2616-8452

**Published:** 2025-06-26

**Authors:** Sho Hasegawa, Shin Yagi, Yusuke Kurita, Yu Honda, Itaru Hashimoto, Kensuke Kubota, Masato Yoneda

**Affiliations:** 1 Department of Gastroenterology and Hepatology, Yokohama City University School of Medicine, Yokohama, Japan; 2 Department of Surgery, Yokohama City University School of Medicine, Yokohama, Japan

In endoscopic ultrasound-guided hepaticogastrostomy (EUS-HGS), tract dilation using dedicated devices is commonly performed prior to stent placement 1 2 3 . Here, we report a rare case of successful one-step placement of a 7-Fr plastic stent in a man in his 50s with obstructive jaundice and minimal intrahepatic bile duct dilation, using EUS-HGS without tract dilation.

The patient had undergone distal gastrectomy with Billroth II reconstruction for recurrent unresectable gastric cancer with multiple lymph node metastases. Six months after the surgery, liver function abnormalities were detected on blood tests. Contrast-enhanced computed tomography (CT) revealed obstructive jaundice caused by enlarged hilar lymph nodes ( Fig. 1 ). Endoscopic retrograde cholangiopancreatography using a single-balloon enteroscope was attempted ( Video 1 ). Although biliary cannulation was successful, the stricture was extremely severe due to the hilar lymph nodes, and the intrahepatic bile ducts could not be visualized. Hence, biliary drainage failed. CT showed minimal dilation of the intrahepatic bile ducts in the left lobe ( Fig. 2 ).

**Fig. 1:**
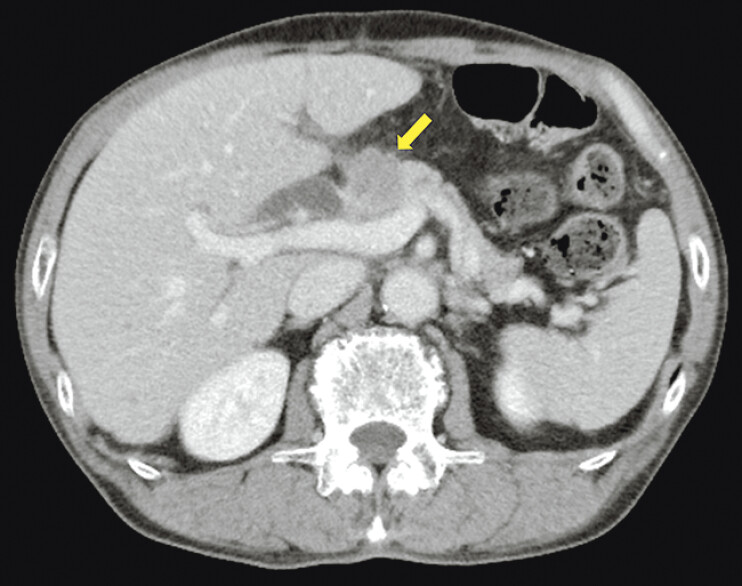
Contrast-enhanced computed tomography showing hilar lymph node metastases (arrow) from the gastric cancer.

**Fig. 2:**
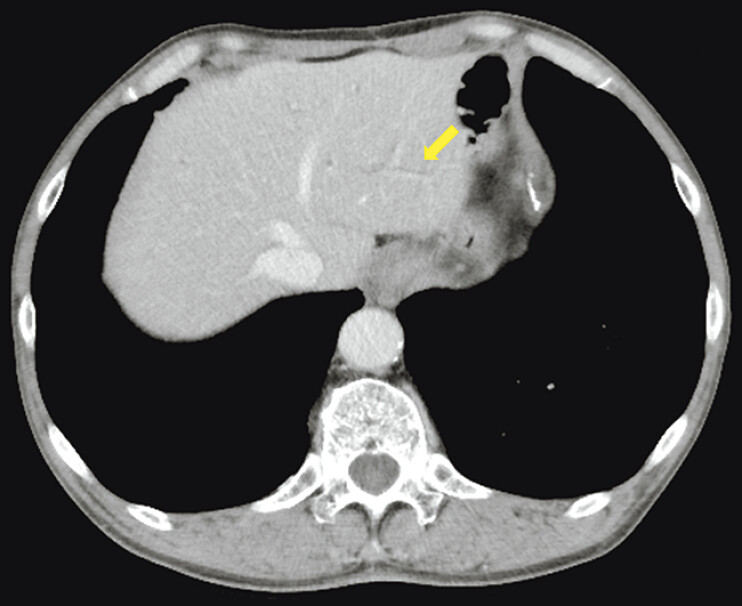
Abdominal computed tomography showing a minimally dilated B2 bile duct (arrow).

Successful plastic stent placement using endoscopic ultrasound-guided hepaticogastrostomy without tract dilation in a case of obstructive jaundice due to metastatic gastric cancer without intrahepatic bile duct dilation.Video 1

EUS-HGS performed the next day revealed that the diameter of the B2 bile duct was 2 mm, indicating poor dilation. Using a 19-gauge fine-needle aspiration needle, the B2 bile duct was punctured, and a guidewire was successfully advanced into it. A cholangiography catheter (SHOREN; Kaneka Medix, Osaka, Japan) was inserted ( Fig. 3 ), bile was aspirated, and contrast was injected. Cholangiography confirmed a complete hilar bile duct obstruction.

**Fig. 3:**
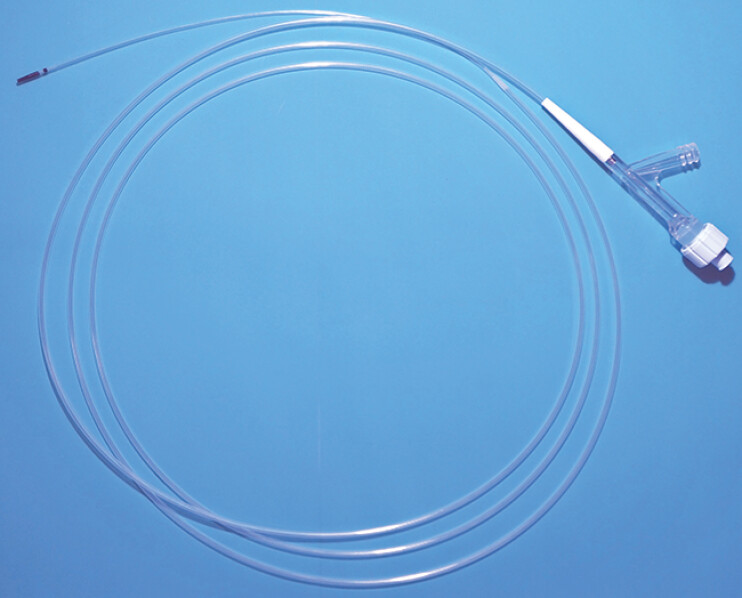
Cholangiography catheter (SHOREN; Kaneka Medix, Osaka, Japan) with a maximum outer diameter of 6.9 Fr.

The SHOREN catheter had an outer diameter of 6.9 Fr, and without the use of a dedicated dilation device, a 7-Fr plastic stent for EUS-HGS (Through & Pass Type-IT; Gadelius Medical, Tokyo, Japan) was successfully deployed. Despite the absence of biliary dilation, the stent was inserted with minimal resistance. CT performed the following day confirmed appropriate stent placement, and no adverse events were noted ( Fig. 4 ).

**Fig. 4:**
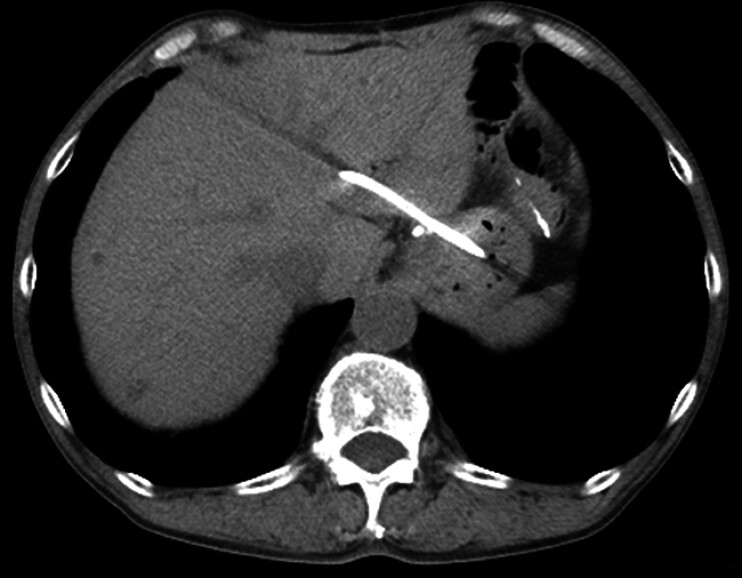
Abdominal computed tomography 1 day after endoscopic ultrasound-guided hepaticogastrostomy showing a plastic stent placed from the stomach to the intrahepatic bile duct.

Endoscopy_UCTN_Code_TTT_1AS_2AD

## Notes

Endoscopy E-Videos https://eref.thieme.de/e-videos
*E-Videos* is an open access online section of the journal *Endoscopy* , reporting on interesting cases and new techniques in gastroenterological endoscopy. All papers include a high-quality video and are published with a Creative Commons CC-BY license. Endoscopy E-Videos qualify for HINARI discounts and waivers and eligibility is automatically checked during the submission process. We grant 100% waivers to articles whose corresponding authors are based in Group A countries and 50% waivers to those who are based in Group B countries as classified by Research4Life (see: https://www.research4life.org/access/eligibility/ ). This section has its own submission website at https://mc.manuscriptcentral.com/e-videos .
